# Deciphering dynamic changes of the aging transcriptome with COVID-19 progression and convalescence in the human blood

**DOI:** 10.1038/s41392-023-01466-9

**Published:** 2023-05-22

**Authors:** Ran Li, Jing Zou, Dongling Pei, Ting Pan, Bing Yang, Xianzhi Liu, Yan Chen, Fangfang Zhou, Long Zhang

**Affiliations:** 1 Hematology Department, The Eighth Affiliated Hospital, Sun Yat-sen University, Shenzhen, 518000 China; 2 MOE Key Laboratory of Biosystems Homeostasis & Protection and Innovation Center for Cell Signaling Network, Life Sciences Institute, Zhejiang University, Hangzhou, 310058 China; 3 Department of Neurosurgery, The First Affiliated Hospital of Zhengzhou University, Zhengzhou, 450001 China; 4 Institute of Human Virology, Zhongshan School of Medicine, Sun Yat-sen University, 510080 Guangzhou, China; 5 Institutes of Biology and Medical Sciences, Soochow University, Suzhou, 215123 China

**Keywords:** Infection, Genome informatics

**Dear Editor**,

Overwhelming evidence suggests that age itself is a prominent risk factor for COVID-19 morbidity and mortality.^1,2^ However, the molecular basis of aging’s effect on SARS-CoV-2 susceptibility and COVID-19 severity in adults is still not fully understood. Thus, we hypothesized that aging-related cellular landscape alterations influence clinical manifestations, which is critical for determining likely intervention targets to slow the transmission of COVID-19 and reduce severe symptoms.

The overall workflow of this study is shown in Fig. 1a. To reveal cell-type-specific characteristics of age-related genes in human peripheral blood, we initially identified multiple gene clusters significantly correlated with age (Supplementary Fig. 1). Among them, 479 genes showed an increase in expression with age (age-pos), while 455 genes decreased in expression with age (age-neg) (Fig. 1b; Supplementary Data S1). Age-pos genes were involved in stress response, cell migration, aging, and immune processes, while age-neg genes were mainly correlated with transcription regulation (Fig. 1c). Moreover, certain age-pos genes were highly expressed in dendritic cells, NK, monocytes, and megakaryocytes, while B cells, CD4^+^ T, and CD8^+^ T cells showed the highly enriched expression of certain age-neg genes (Supplementary Fig. 2, 3a, b and Data S1). For example, among the NK cell-enriched age-pos genes, *KLRD1* and *CCL4* showed an increase in expression with age, while among the CD4^+^ T cell-enriched age-neg genes, *LEF1* and *CCR7* decreased in expression with age (Supplementary Fig. 3c, d).

**Fig. 1:**
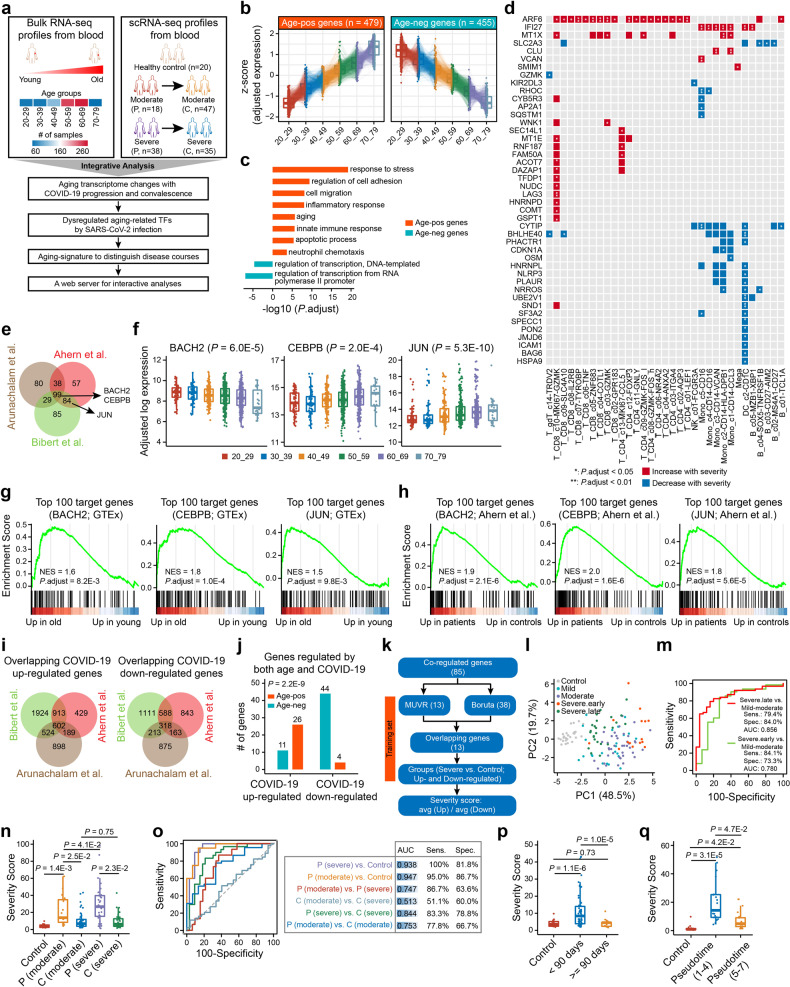
Main results of this study. **a** Workflow for this study. P progression, C convalescence. **b** The relative expression of age-related gene among different age groups in the GTEx dataset. **c** DAVID gene ontology (GO) analysis of age-pos genes (orange) and age-neg genes (blue). **d** Heatmap showing the cell-types with significant difference in proportion of cells expressing certain age-pos genes among patients with different severity during progression stages. Significance tests were performed by two-sided Kruskal-Wallis test and *p*-values were adjusted using the Benjamini & Hochberg method. Data are from GSE158055.^3^ **e** Venn diagram showing the intersections of age-related genes that were regulated by SARS-CoV-2 infection from three independent studies.^4–6^ **f** Comparison of aging-related transcription factors expression among different age groups. Significance tests were performed by two-sided Kruskal-Wallis test. **g**, **h** GSEA analysis indicating top 100 putative target genes of three aging-related transcription factors were significantly upregulated in old healthy adults (**g**) and severe patients (**h**).^4^ NES, normalized enrichment score. **i** Venn diagram showing the intersections of COVID-19 upregulated genes (left), and the intersections of COVID-19 downregulated genes (right) from three independent studies.^4–6^ **j** The intersections between the directionality of change in age-related gene expression with age and the directionality of change in COVID-19 regulated genes expression with infection onset. Significance test was performed by two-tailed Fisher’s exact test. **k** Workflow for this study to develop the severity scoring model. **l** Principal component analysis (PCA) showing obvious separation among patient groups with different severity based on the severity score.^5^ Severe.early, patients were sampled within the first 7 days in hospital; Severe.late, patients were sampled later. **m** ROC curve analyses to evaluate performance of the model in differentiating patient groups.^5^ **n**, **o** Boxplot (**n**) and ROC curves (**o**) showing the difference of the severity score among patient groups with different severity and stages in one validation dataset. Significance tests were performed by two-tailed Student’s *t*-test. Data are from GSE158055.^3^ **p** Association between the severity score and sampling days (days after symptom onset) of patients in convalescence stages. Significance tests were performed by two-tailed Student’s *t*-test. Data are from GSE158055.^3^ **q** Boxplot showing the significant difference of severity score among healthy controls and patients with different disease trajectory pseudotimes in another validation dataset. Significance tests were performed by two-tailed Student’s *t*-test. Data are from GSE161777^7^

Using a large scRNA-seq dataset of 159 PBMC samples,^3^ we discovered a significant association between age-related scores and COVID-19 severity, stage, patient age, and sampling time (days after symptom onset; the same applies to the full article) (Supplementary Fig. 4a-c and Data S2). We only included age- and sample-type-matched patients in progression stages and healthy controls, and observed that age-pos genes were upregulated in severe patients, while age-neg genes were significantly downregulated (Supplementary Fig. 4d). Furthermore, age-related score increased (ssGSEA-pos score) or decreased (ssGSEA-neg score) progressively from healthy controls to mild-moderate to severe patients (Supplementary Fig. 4e). These results indicated age-dependent effects in the susceptibility and progression of SARS-CoV-2 infection.

We then examined changes in the proportion of cells expressing certain age-related genes in patients with different severity during progression stages. We focused on a subset of age-pos genes that were highly expressed across cell-types (Supplementary Fig. 5a and Data S3). In one T cell cluster (T_CD8_c10-MKI67-GZMK), the proportion of cells expressing these genes increased with disease severity during the progression stage. However, it decreased significantly during convalescence and returned to healthy control levels with increased recovery time (Supplementary Fig. 5b, 6a). Conversely, CD1c^+^ DCs and CD16^+^ monocytes showed a marked decrease in severe patients (Supplementary Fig. 5b, 6b, c), and the proportion was positively correlated with sampling time during convalescence stage (Supplementary Fig. 6d). We identified 44 age-pos genes associated with COVID-19 severity in 33 cell-types (Fig. 1d), with 43.2% of genes increasing in proportion with disease severity. For example, the proportion of two monocyte subtypes (Mono_c1-CD14-CCL3 and Mono_c3-CD14-VCAN) expressing *CLU* increased progressively from healthy controls to severe patients, but decreased during convalescence. And 50 days after symptom onset, the proportion returned to levels comparable to healthy controls (Supplementary Fig. 6e-g). We also found 12 age-neg genes in 11 cell-types, for example, the proportion of naive CD8^+^ T cells expressing *BACH2* or *ABLIM1* was significantly decreased in severe patients and then elevated in late convalescence (Supplementary Fig. 7).

The critical mechanisms underlying post-acute COVID-19 syndrome remain elusive. To this end, a cohort of COVID-19 patients during convalescence stage who experienced severe symptoms was selected.^3^ We identified 24 age-pos genes and 12 age-neg genes with significant correlation to sampling time (Supplementary Fig. 8a-d). Expression of these genes was comparable in late-sampled convalescent individuals and healthy controls. Most of these age-pos genes was frequently expressed in dendritic cells (Supplementary Fig. 8e). *LEPROTL1* was enriched in several cell-types, especially CD8^+^ and CD4^+^ T cells (Supplementary Fig. 8f). Similar results were found in individuals who survived moderate symptoms (Supplementary Fig. 8a, b). These findings suggest persistent age-related expression profile alterations during convalescent stage.

Next, we focused on aging-related transcription factors (TFs) modulated by SARS-CoV-2 infection. Multiple independent studies showed significant overlap between age-related genes and COVID-19-regulated genes^4–6^ (Supplementary Fig. 9a, b and Data S4). We found significant consistency in the direction of expression changes between age-related genes and COVID-19 regulated genes (Supplementary Fig. 9c), suggesting dysfunction of age-related genes with the onset of SARS-CoV-2 infection. Among a consensus set of genes (Fig. 1e; Supplementary Fig. 10), we focused on three TFs, including *BACH2*, *CEBPB* and *JUN*, whose activity was perturbed by both age and SARS-CoV-2 infection. Aging and SARS-CoV-2 infection decreased expression of *BACH2*, while expression of *CEBPB* and *JUN* was increased in both older adults and patients (Fig. 1f; Supplementary Fig. 11a, b). We collected the top 100 putative target genes for each TF (Supplementary Data S4). GSEA showed that the target genes of all three TFs were significantly enriched in older adults (Fig. 1g), indicating increased activity of *JUN* and *CEBPB* as transcriptional activators with age and decreased activity of *BACH2* as a transcriptional repressor. Upregulation of TF target genes was also observed in COVID-19 patients (Fig. 1h), suggesting that changes in the activity of *BACH2*, *JUN*, and *CEBPB* during aging might contribute to SARS-CoV-2 susceptibility. These results confirmed by multiple independent studies (Supplementary Fig. 11c). Additionally, *CEBPB* expression in monocyte subsets and *JUN* expression in almost all cell subsets were significantly correlated with severity during the progression stage (Supplementary Fig. 11d-f).

We further built an age-related signature linked to COVID-19 severity and trajectory. Using three COVID-19 datasets,^4–6^ we identified 602 upregulated overlapping genes in patients, of which 26 increased and 11 decreased in expression with age (Fig. 1i, j). We also found 318 downregulated overlapping genes, including four in the age-pos group and 44 in the age-neg group (Fig. 1i, j). SARS-CoV-2 infection shares a certain degree of similarity with Influenza virus in transcriptional changes, but not with Zika virus (Supplementary Fig. 12). The 13 of these 85 co-regulated genes were selected by two variable selection algorithms to develop the severity scoring model, which effectively discriminated patients with different severity in training set^5^ (Fig. 1k-m; Supplementary Fig. 13). In an independent validation cohort,^3^ the model also showed excellent performance in discriminating patients in the progression stages from those in the convalescence stages (Fig. 1n, o). In the progression stage, patients with severe symptoms had higher scores than those with moderate symptoms (Fig. 1n, o). The severity score of patients in convalescence stages was correlated with sampling time (Fig. 1p). In another validation cohort,^7^ patients were grouped according to disease trajectory pseudotimes. As expected, patients in incremental phase to the early convalescence (pseudotime 1–4) had the highest severity scores, while the score was decreased during late convalescence and long-term follow-up (pseudotime 5–7) (Fig. 1q).

To facilitate better utilization of the current data resources, we introduced a web server named scAgCov (http://longlab-zju.cn/scAgCov/) for users to query age-related genes and compare their expression in a given cell-type among COVID-19 patients with different severity and stages.

In summary, our study differs from previous studies^8–10^ on age correlation in COVID-19 patients due to its unique biological problem and research methodology. We focused on identifying age-related genes linked to COVID-19 severity in specific cell-types, using a larger scRNA-seq dataset. Our established severity scoring model holds the potential to evaluate the risk of post-acute COVID-19. Additionally, we introduced a web server to facilitate researchers in accessing this valuable resource. However, these findings are based on public resource and require further validation through wider experiments. Nonetheless, we hope this study can inform clinical decision-making to develop personalized therapies for preventing sequela and mortality.

## Supplementary information

Supplementary Materials

Supplementary Data S1

Supplementary Data S2

Supplementary Data S3

Supplementary Data S4

## Data Availability

The raw data relating to the current study are freely available from the links described in the sequencing data and clinical phenotyping acquisition section of the Methods. The processed data supporting the key findings of this paper are available in the article and in its online supplementary information files or from the corresponding author upon reasonable request.
