# Supplementary material for: Deciphering dynamic changes of the aging transcriptome with COVID-19 progression and convalescence in the human blood
Source: Signal Transduct Target Ther. 2023 May 22;8:206. doi: 10.1038/s41392-023-01466-9 (PMC10200789; doi:10.1038/s41392-023-01466-9)
Supplement: Supplementary file 1 — Supplementary Materials [file 41392_2023_1466_MOESM1_ESM.docx]

**Supplementary Materials for**

**Deciphering dynamic changes of the aging transcriptome with COVID-19 progression and convalescence in the human blood**

Ran Li^1*^, Jing Zou^2*^, Dongling Pei^3*^, Ting Pan^4*^, Bing Yang^2^, Xianzhi Liu^3^, Yan Chen^1 ¶^, Fangfang Zhou^5 ¶^, Long Zhang^1,2 ¶^

*Equal contributions

^¶^Correspondence should be addressed to: L.Z. ([L_Zhang@zju.edu.cn](mailto:L_Zhang@zju.edu.cn)), F.Z. ([zhoufangfang@suda.edu.cn](mailto:zhoufangfang@suda.edu.cn)) or Y.C. (cheny658@mail.sysu.edu.cn)

**This PDF file includes:**

Materials and Methods

Supplementary Text

Figures. S1 to S13

Captions for Data S1 to S4

**Other Supplementary Materials for this manuscript include the following:**

Data S1 to S4

Data S1. (separate file): List of age-related genes and cell-type characteristics

Data S2. (separate file): Unadjusted and adjusted ssGSEA score

Data S3. (separate file): Cell-type expression of age-related genes in different patient groups

Data S4. (separate file): Differential expression analysis between healthy controls and COVID-19 patients, list of age-related genes that were perturbed by SARS-CoV-2 infection, and top 100 putative target genes for transcription factor

**Materials and Methods**

***Sequencing data and clinical phenotyping acquisition***

The blood RNA-seq raw counts and normalized expression profiles (Transcripts Per Kilobase Million, TPM) of 755 donors were collected from the Genotype-Tissue Expression (GTEx) Portal (https://gtexportal.org/home/datasets). Single-cell transcriptomes and clinical characteristics of 158 human PBMCs samples, including 20 healthy controls, 56 patients during progression stages (18 with mild-moderate symptoms and 38 with severe symptoms) and 82 recovered convalescent persons (47 with mild-moderate symptoms and 35 with severe symptoms), were download from Gene Expression Omnibus (Ren et al., Cell 2021, GSE158055)^1^. Two single cell transcriptomes of PBMCs based on 3’ and 5’ methods as reference matrices to estimate cell type proportions were obtained from CIBERSORTx (Newman et al., Nat Biotechnol 2019, https://cibersortx.stanford.edu/download.php)^2^. Bulk transcriptomes and clinical characteristics of patients with SARS-CoV-2 infection and healthy controls were collected from four independent studies: (1) healthy controls (n = 27), mild COVID-19 (n = 23), moderate COVID-19 (n = 40), early severe COVID-19 (sampled within the first 7 days in hospital, n = 15) and late severe COVID-19 (sampled later, n = 25) (Bibert et al., Front Immunol 2021, https://data.mendeley.com/datasets/8wxhhykfnh/2)^3^; (2) healthy controls (n = 17) and COVID-19 patients (n = 12) (Arunachalam et al., Science 2020, GSE152418)^4^; (3) healthy controls (n = 27), mild COVID-19 (n = 18), severe COVID-19 (n = 41) and critical COVID-19 (n = 19) (Ahern et al., Cell 2022, https://doi.org/10.5281/zenodo.6120249)^5^; (4) Patients were grouped according to disease trajectory pseudotimes, including 14 samples in incremental phase, 19 samples in the early convalescence and 23 samples in late convalescence and long-term follow-up (Bernardes et al., Immunity 2020, GSE161777)^6^. We also collected single cell transcriptomes of PBMCs from two studies in relation to human aging: (1) young (n = 3; age, 30.7 ± 10.0 years) and old healthy adults (n = 6; age, 85.8 ± 11.1 years) from Gene Expression Omnibus (Luo et al., Nature Aging, GSE157007)^7^; (2) young healthy adults (n = 8, age, 20-45 years) and aged healthy adults (n = 8, ≥60 years) from the National Genomics Data Center (Zheng et al., Protein Cell 2020, HRA000203)^8^. Two other bulk datasets associated with aging can be found at dbGaP (Márquez et al., Nat Commun 2020, ID: phs001934.v1.p1)^9^.

***Identifying age-related genes based on bulk transcriptomes of human blood***

The age-related genes were identified using the likelihood-ratio test (LRT) model in R package DESeq2^10^ (v1.28.1). Two age-independent clinical variables including sex and Hardy scale were controlled in the LRT model. Statistical significance threshold of BH adjusted *p* < 0.05 was used to obtain differentially expressed genes among age groups, and z-score normalization for these gene expression. We then utilized the degPatterns function in the R package DEGreport (v1.24.1) to make groups of age-related gene based on z-score normalized expression profiles. Gene groups that the expression had progressively increased (age-pos) or decreased (age-neg) trends with age were retained for subsequent analysis. Gene ontology (GO) enrichment analysis were performed for age-pos and age-neg genes using DAVID database^11^ (v6.8). For visualization of adjusted age-related genes expression, a variance stabilizing transformation (VST) function in DESeq2 was first applied to the count data, then the sex and Hardy scale as covariates were adjusted using removeBatchEffect function in limma^12^ (v3.44.3).

***Determining cell-type abundance of the human blood***

To assess the change in cell-type proportion with age, the CIBERSORTx tool^2^ was utilized to estimate cell-type abundance based on the bulk transcriptomes of human blood from the GTEx. Specifically, the PBMC single-cell reference matrix from 10x platform was provided as input of CIBERSORTx, and the S-model batch correction was applied to estimate cell-type proportion for each blood sample. These results were used to perform the following analyses: (1) changes in the proportions of blood cell types with age. Only cell types with non-zero estimated proportions in more than 50% of samples were retained for follow-up analysis. To visualize using forest plot, an ordinal logistic regression model, which was implemented in R package MASS and ordinal, was used to estimate coefficients and statistical significance, and sex and Hardy scale were incorporated in this model. Statistical significance was further adjusted by BH correction. The 95% confidence intervals of estimated coefficients were presented in forest plot; (2) comparison of cell-type proportions among all age groups. The estimated cell-type proportions were adjusted using residuals from a generalized linear model that incorporated sex and Hardy scale as covariates.

***Single-cell RNA-sequencing data analysis***

The R package Seurat^13^ (v4.1.0) was used to analyze single-cell whole-transcriptome gene expression. Three quality control metrics were used for data processing, including the total UMI counts, number of detected genes and proportion of mitochondrial gene per cell. The detailed parameters and cell type annotations referred to the literature of data sources. The processed data was used for following analyses: (1) exploring the difference in proportion of cells expressing a given gene among patient groups. A threshold of UMI count > 0 was used for expressed gene in each cell. Percentage of cells within a cell type expressing a given gene was estimated by dividing cells with UMI count > 0 by all cells; (2) comparison of a given gene expression among patient groups. The expression count data for genes detected in each sample were normalized using NormalizeData function applied in Seurat; (3) identification of COVID-19 regulated genes or differential expression analysis based on bulk transcriptomes. To obtain pseudo-bulk profiles from single-cell transcriptomes, we summed the UMI counts for each gene of all cells from each sample.

***Single sample Gene Set Enrichment Analysis (ssGSEA)***

The ssGSEA was performed using R package GSVA^14^ (v1.36.3). The 479 age-pos genes and 455 age-neg genes as two prior defined sets of genes were used to calculate enrichment score for each sample. Then, the sex and sample type as covariates to adjust enrichment score using removeBatchEffect function. Patients were grouped based on clinical variables, and statistical significance of adjusted ssGSEA score difference among patient groups was calculated by two-sided Kruskal-Wallis test and adjusted using the Benjamini-Hochberg (BH) correction.

***Differential expression analysis between patients and controls***

The DESeq2 was used to examine whether genes were differentially expressed between COVID-19 patients and healthy controls. We adjusted the statistical significance using BH correction. In each pairwise comparison, the following cut-off criteria were used to define differential genes: (1) BH adjusted *p* value should be less than 0.05; (2) fold change (expressed as ratio of gene expression in patients versus healthy controls) ≤ -log2 (1.5) for downregulation or fold change (expressed as ratio of gene expression in patients versus healthy controls) ≥ log2 (1.5) for upregulation in patients. If clinical information including age and sex were available, age and sex as covariates were controlled in the LRT model, otherwise we used the Wald significance test in DESeq2 to select differentially expressed genes.

***Transcriptional factor analysis***

Here, we briefly described the procedure of selecting transcription factor (TF) that was associated with aging and COVID-19 regulation: (1) TF was an age-related gene; (2) TF was significantly differential expression (BH adjusted *p* < 0.05 and |Log2 (FC)| > log2 (1.5)) between COVID-19 patients and healthy controls in at least two independent studies; (3) the directionality of COVID-19 regulation was the same in above independent studies. To further confirm alteration in activity of selected TFs in the old adults and COVID-19 patients, we collected the top 100 putative target genes for each TF from Cistrome platform (http://cistrome.org/db/#/). The OCI-Ly7 cell line derived from human blood was used to query the *BACH2* targets^15^. The THP-1 cell line derived from human blood was used to query the *CEBPB* targets^16^. The Calu-3 cell line derived from human lung was used to query the *JUN* targets^17^. Then, GSEA was performed using R package clusterProfiler^18^ (v3.12.0), in this analysis, fold change of gene expression in patients versus healthy controls or old adults versus young adults was used as input, and target genes of TF as priori defined set of genes.

***Construction and validation of patient groups predictor by multiple machine learning methods***

We utilized two algorithms to select suitable variables: (1) MUVR^19^ (v0.0.975), a R package that allows for predictive multivariate modeling with minimally biased variable selection incorporated into a repeated double cross-validation (CV) framework. Random Forest core modeling and 1000 repetitions of double CV were used, and all other parameters with default; (2) Boruta^20^ (v7.0.0), a wrapper built around the Random Forest classification algorithm that reports the important and interesting features with respect to an outcome variable. The 1000 importance source runs were used, and all other parameters with default. The important genes selected by both MUVR and Boruta were used to develop the severity scoring model represented as metagene scores, which was calculated as the ratio of average expression of upregulated genes to the average expression of downregulated genes referred to previous published method. Upregulated and downregulated genes were defined according to the groups. For example, differential expression analysis was performed between severe groups and healthy controls, we used upregulated or downregulated genes in the severe groups. To evaluate and validate the performance of severity score based on a set of genes, we performed receiver operating characteristic (ROC) curve analyses to determine the area under the ROC curve (AUC), sensitivity and specificity for each pairwise comparison. The ROC analysis and visualization was conducted using R package pROC (v1.18.0) and plotROC (v2.3.0). Principal component analysis (PCA) was performed using prcomp function in R package stats (v4.0.02).

***Functional enrichment analysis***

Functional annotation for genes of interest, including Biological processes (BP), Cellular component (CP) and Molecular function (MF), was performed by using DAVID database. The *P* < 0.05 was considered statistically significant. The protein-protein interaction (PPI) network was conducted using STRING database^21^ with default parameters, and then interaction information as input for Cytoscape^22^ (v3.8.2) to visualize network.

***Batch effect correction***

In bulk transcriptome data, ComBat function in R package sva^23^ (v3.36.0) was used to remove batch effects. For scRNA-seq data, we utilized the harmony algorithm^24^ to do batch effect correction.

***Developing the web server***

The R package Shiny^25^ (v1.6.0) was used to build a website for the interactive visualization of the data from this study. At present, the web server features were divided into three major tabs, including Age-related Genes, Cell-type Percentage and Cell-type Expression. Plots were generated using ggplot2^26^. Statistics for box plots were calculated using the Kruskal–Wallis test. The two-sided Student’s t test was utilized for pairwise groups comparison.

***Statistical analysis***

Statistical details of analyses can be found in the figure legends and main text above. Statistical significance tests, including the Kruskal-Wallis test, hypergeometric test, Fischer’s exact test, Student’s t test and Pearson correlation test in this study, were performed by using R software (v4.0.2). Statistical significance was adjusted using the Benjamini-Hochberg (BH) FDR correction. All statistical tests were two-sided, and the significance threshold was considered as *p* value < 0.05 or BH adjusted *p* value < 0.05. For box plot, the box and line inside box indicated interquartile range (IQR) and median, respectively, the whiskers indicated points within Q3 + (1.5x IQR) and Q1 - (1.5x IQR), of these, Q1 and Q3 represented first and third quartiles, respectively. Data in the bar plot were presented as mean ± SEM.

***Data availability***

The raw data relating to the current study are freely available from the links described in the Sequencing data and clinical phenotyping acquisition section of the Methods. The processed data supporting the key findings of this paper are available in the article and in its online supplementary information files or from the corresponding author upon reasonable request.

***Code availability***

The code for data analyses in this study have been deposited to GitHub (https://github.com/ranlibio/scAgCov).

**Supplementary Text**

***The uniqueness of this study***

Here, we analyzed blood samples using multi-omics mapping to study the relationship between age-related single-cell transcriptomic profile changes and SARS-CoV-2 infection, disease progression, and convalescence in adults. Our findings suggested potential targets for treating severe symptoms. Compared to previous studies on age correlation in COVID patients^8,27,28^, our study presented the uniqueness in the biological problem addressed and the research methodology employed.

Firstly, we utilized the bulk transcriptome of peripheral blood to define age-related genes. The large cohort of 755 different donors with multiple age groups (n = 6) allowed us to identify clusters of genes in which their expression progressively changes with age, rather than a simple comparison between two age groups. Secondly, to identify key aging-related genes expressed in specific cell types with COVID-19 severity, we used the largest scRNA-seq dataset (159 PBMC samples)^1^ reported so far, which includes multiple disease stages. In contrast, Zheng et al.^8^ focused on investigating the impact of aging on COVID-19 onset and recovery. Thirdly, we developed an age-related signature linked to COVID-19 severity and trajectory by utilizing 13 co-regulated genes, which performed well in discriminating patients with different severity and stages. Finally, we introduced a web server that would facilitate researchers to compare age-related gene expression among patients with different severity in a given cell type. These efforts should have both scientific meaning and values for translational applications.

**Supplemental references**

1 Ren, X. *et al.* COVID-19 immune features revealed by a large-scale single-cell transcriptome atlas. *Cell*. **184**, 1895-1913 e1819, (2021).

2 Newman, A. M. *et al.* Determining cell type abundance and expression from bulk tissues with digital cytometry. *Nat Biotechnol*. **37**, 773-782, (2019).

3 Bibert, S. *et al.* Transcriptomic Signature Differences Between SARS-CoV-2 and Influenza Virus Infected Patients. *Front Immunol*. **12**, 666163, (2021).

4 Arunachalam, P. S. *et al.* Systems biological assessment of immunity to mild versus severe COVID-19 infection in humans. *Science*. **369**, 1210-1220, (2020).

5 Ahern, D. J. *et al.* A blood atlas of COVID-19 defines hallmarks of disease severity and specificity. *Cell*. **185**, 916-938 e958, (2022).

6 Bernardes, J. P. *et al.* Longitudinal Multi-omics Analyses Identify Responses of Megakaryocytes, Erythroid Cells, and Plasmablasts as Hallmarks of Severe COVID-19. *Immunity*. **53**, 1296-1314 e1299, (2020).

7 Luo, O. J. *et al.* Multidimensional single-cell analysis of human peripheral blood reveals characteristic features of the immune system landscape in aging and frailty. *Nature Aging*. **2**, 348-364, (2022).

8 Zheng, Y. *et al.* A human circulating immune cell landscape in aging and COVID-19. *Protein Cell*. **11**, 740-770, (2020).

9 Marquez, E. J. *et al.* Sexual-dimorphism in human immune system aging. *Nat Commun*. **11**, 751, (2020).

10 Love, M. I., Huber, W. & Anders, S. Moderated estimation of fold change and dispersion for RNA-seq data with DESeq2. *Genome Biol*. **15**, 550, (2014).

11 Sherman, B. T. *et al.* DAVID: a web server for functional enrichment analysis and functional annotation of gene lists (2021 update). *Nucleic Acids Res*, (2022).

12 Ritchie, M. E. *et al.* limma powers differential expression analyses for RNA-sequencing and microarray studies. *Nucleic Acids Res*. **43**, e47, (2015).

13 Butler, A. *et al.* Integrating single-cell transcriptomic data across different conditions, technologies, and species. *Nat Biotechnol*. **36**, 411-420, (2018).

14 Hanzelmann, S., Castelo, R. & Guinney, J. GSVA: gene set variation analysis for microarray and RNA-seq data. *BMC Bioinformatics*. **14**, 7, (2013).

15 Swaminathan, S. *et al.* BACH2 mediates negative selection and p53-dependent tumor suppression at the pre-B cell receptor checkpoint. *Nat Med*. **19**, 1014-1022, (2013).

16 Heinz, S. *et al.* Transcription Elongation Can Affect Genome 3D Structure. *Cell*. **174**, 1522-1536 e1522, (2018).

17 Fossum, S. L. *et al.* Ets homologous factor (EHF) has critical roles in epithelial dysfunction in airway disease. *J Biol Chem*. **292**, 10938-10949, (2017).

18 Wu, T. *et al.* clusterProfiler 4.0: A universal enrichment tool for interpreting omics data. *Innovation (Camb)*. **2**, 100141, (2021).

19 Shi, L. *et al.* Variable selection and validation in multivariate modelling. *Bioinformatics*. **35**, 972-980, (2019).

20 Kursa, M. B. & Rudnicki, W. R. Feature Selection with the Boruta Package. *Journal of Statistical Software*. **36**, 1 - 13, (2010).

21 Szklarczyk, D. *et al.* STRING v11: protein-protein association networks with increased coverage, supporting functional discovery in genome-wide experimental datasets. *Nucleic Acids Res*. **47**, D607-D613, (2019).

22 Shannon, P. *et al.* Cytoscape: a software environment for integrated models of biomolecular interaction networks. *Genome Res*. **13**, 2498-2504, (2003).

23 Leek, J. T. *et al.* The sva package for removing batch effects and other unwanted variation in high-throughput experiments. *Bioinformatics*. **28**, 882-883, (2012).

24 Korsunsky, I. *et al.* Fast, sensitive and accurate integration of single-cell data with Harmony. *Nat Methods*. **16**, 1289-1296, (2019).

25 Chang, W. *et al.* Shiny: web application framework for R. *R package version 1*, <http://CRAN.R-project.org/package=shiny>, (2017).

26 Wickham, H. *ggplot2: Elegant Graphics For Data Analysis*. (Springer, 2016).

27 Chow, R. D., Majety, M. & Chen, S. The aging transcriptome and cellular landscape of the human lung in relation to SARS-CoV-2. *Nat Commun*. **12**, 4, (2021).

28 Peters, M. J. *et al.* The transcriptional landscape of age in human peripheral blood. *Nat Commun*. **6**, 8570, (2015).

29 Carlin, A. F. *et al.* A longitudinal systems immunologic investigation of acute Zika virus infection in an individual infected while traveling to Caracas, Venezuela. *PLoS Negl Trop Dis*. **12**, e0007053, (2018).


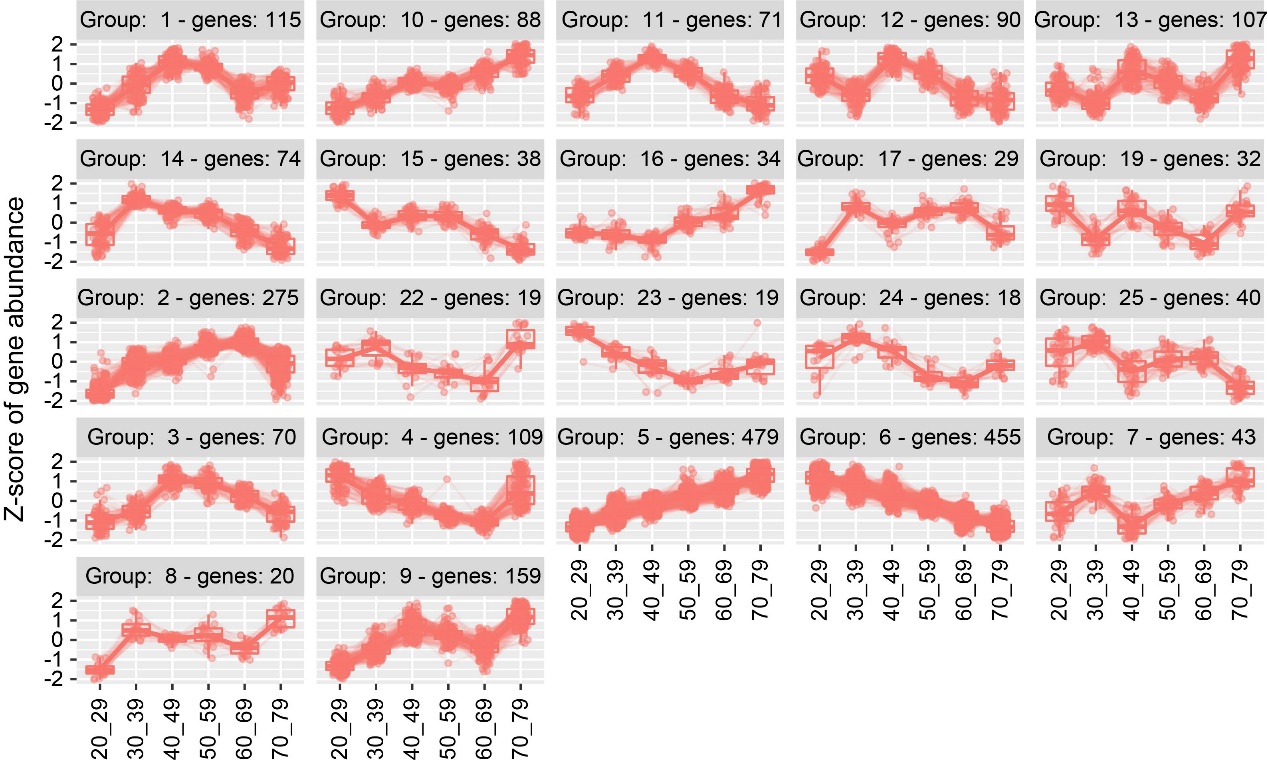


**Supplementary Figure 1.** Differentially expressed genes identified by DESeq2 (BH adjusted p < 0.05) were grouped based on expression profiles. Data are from the GTEx dataset.


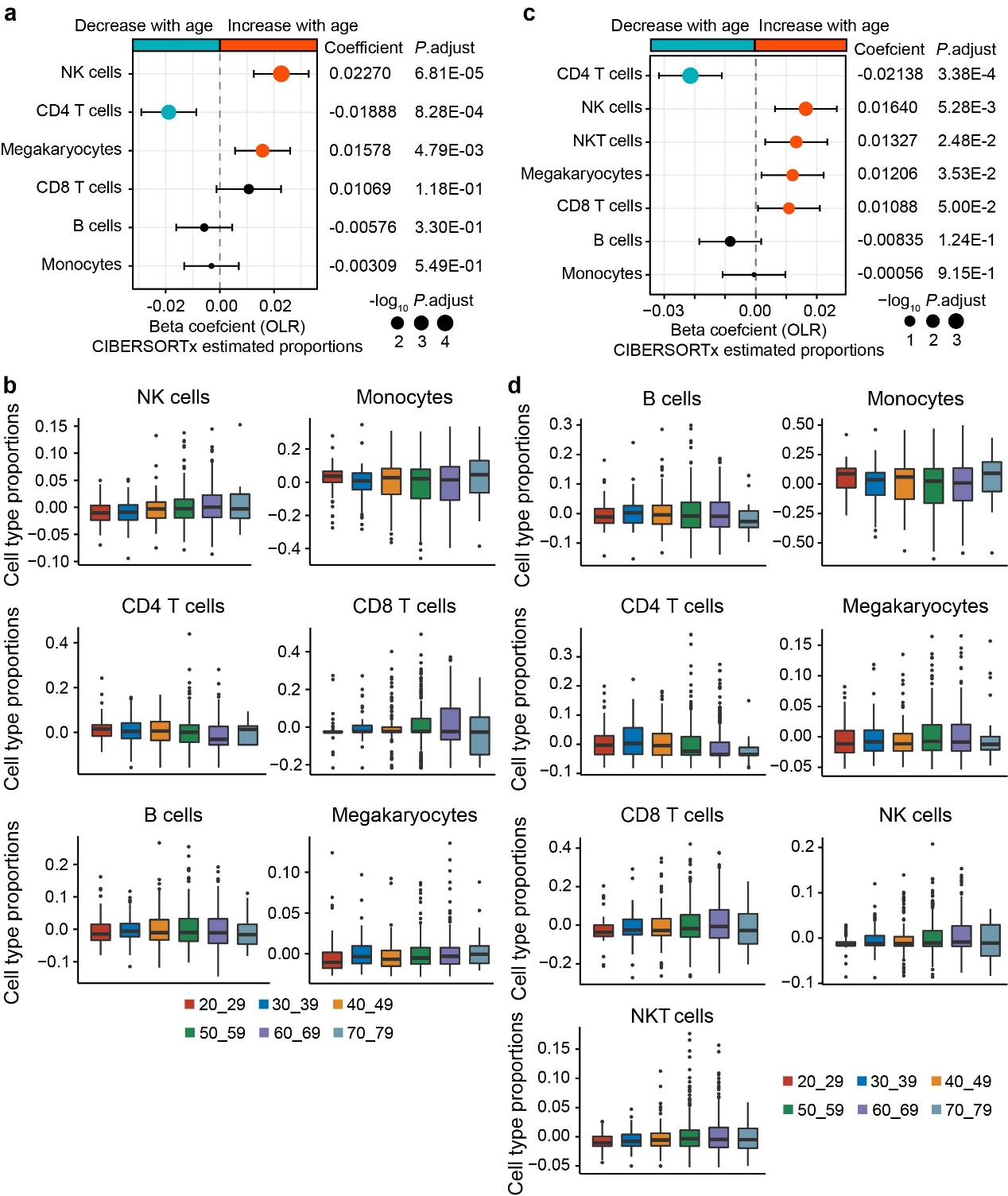


**Supplementary Figure 2. Association of cellular compositions with age. (a, c)** Forest plot of age-related changes in the proportions of blood cell types. Only the cell types with non-zero estimated proportions in > 50% of samples were retained for subsequent analyses. Positive coefficient represents cell types increase in proportion with age, while negative coefficient indicates cell types decrease in proportion with age. By controlling for sex and Hardy scale, statistical significance was calculated using a nonparametric ordinal logistic regression model and further adjusted using the Benjamini-Hochberg (BH) correction. Error bars indicate 95% confidence intervals. **(b, d)** Comparison of adjusted cell-type proportions among different age groups. The proportions were adjusted using residuals from a generalized linear model that incorporated sex and hardy scale as covariates. The cell-type signature matrix was generated using scRNA-seq profiles of healthy 5' **(a, b)** and healthy 3' **(c, d)** PBMCs^2^.


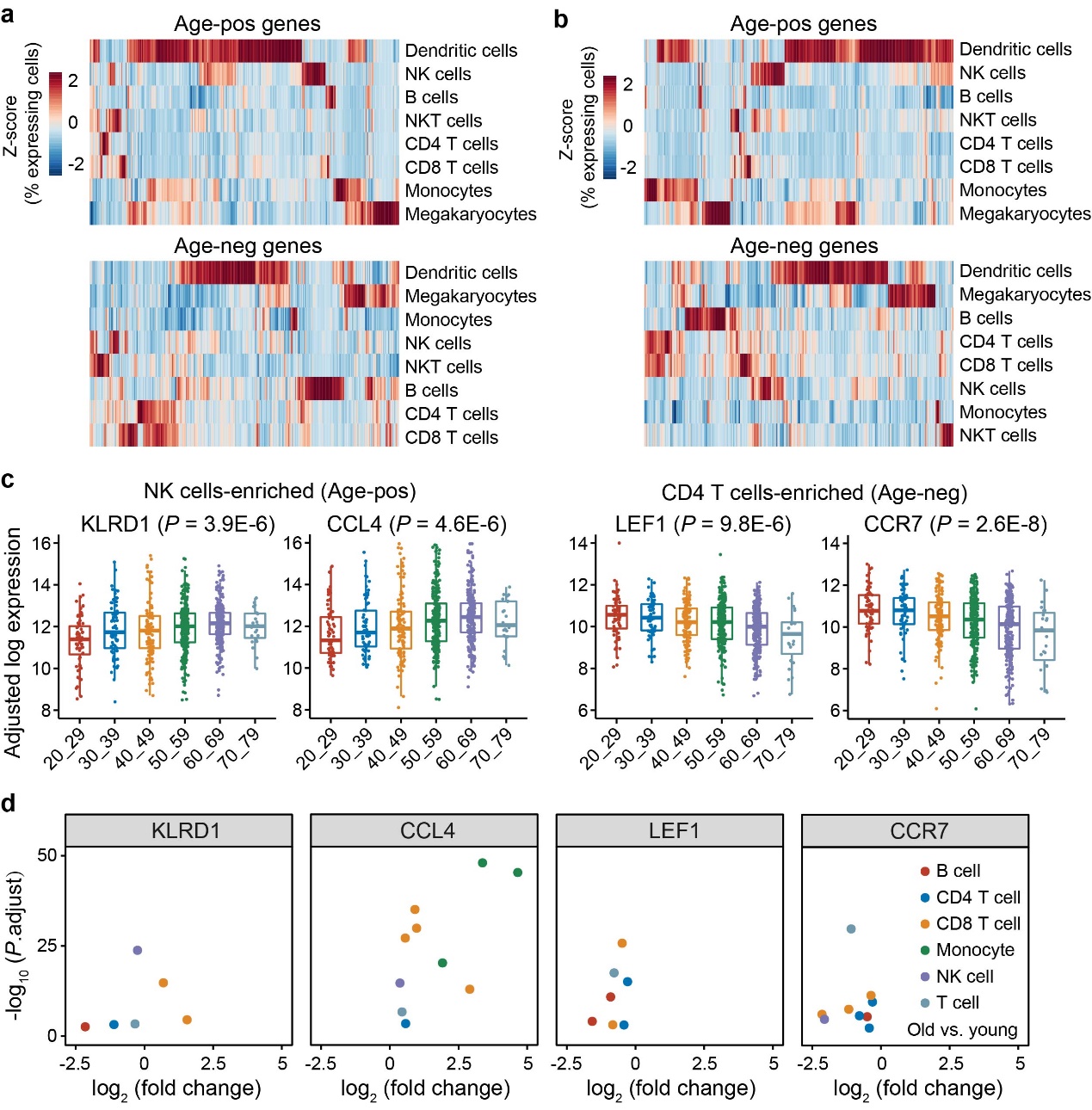


**Supplementary Figure 3. Cell-type-specific characteristic of age-related gene.** **(a, b)** Normalized proportion of cells expressing each of the age-pos genes (top) and age-neg genes (bottom) in two independent datasets, scaled in the column direction. Data are from scRNA-seq profiles of healthy 5' **(a)** and 3' **(b)** PBMCs^2^. **(c)** Comparison of *KLRD1*, *CCL4*, *LEF1* and *CCR7* expression among different age groups. Expression were estimated by controlling sex and Hardy scale. Statistical analysis was performed by two-sided Kruskal-Wallis test. Data are from the GTEx dataset. **(d)** Comparison of *KLRD1*, *CCL4*, *LEF1* and *CCR7* expression of main cell-types from human PBMCs between young healthy adults and aged healthy adults. Data was obtained from an Aging Atlas Consortium (https://ngdc.cncb.ac.cn/aging/index) to validate the result in **(c)**.


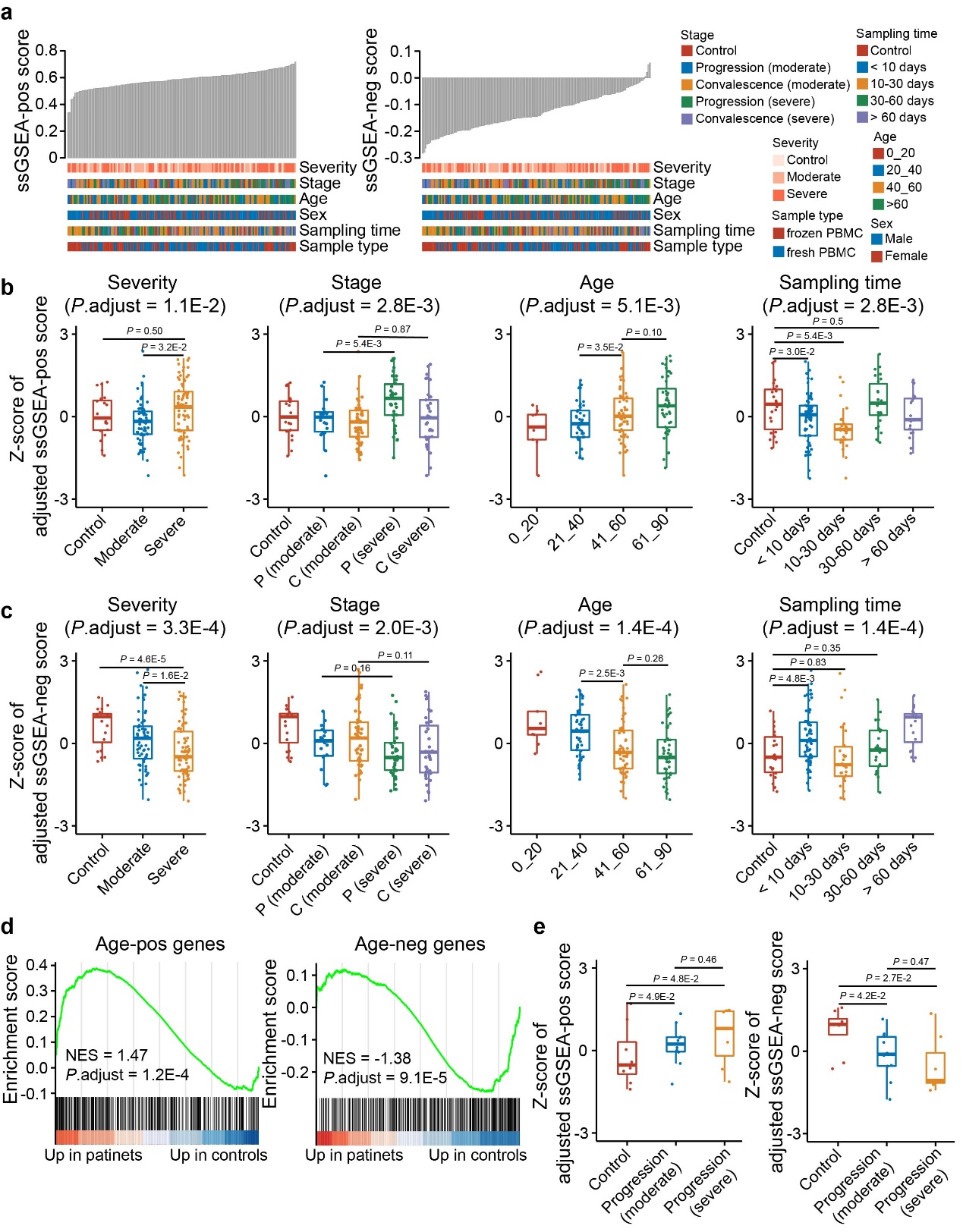


**Supplementary Figure 4. Association of age-related gene expression profiles with clinical variables of COVID-19 patients. (a)** An overview of the association between unadjusted ssGSEA score and clinical pathological variables based on 158 PBMCs samples. Columns represent samples ranked by ssGSEA score from low to high, and rows represent available clinical variables of COVID-19 patients. The score was calculated based on age-pos genes (left) and age-neg genes (right), respectively. **(b, c)** Comparison of adjusted ssGSEA score among patient groups. Patients were grouped by patient severity, stage, age and sampling time. The score was estimated based on age-pos genes (b) or age-neg genes (c) by controlling for sample type (fresh or frozen PBMCs) and sex (male or female). **(d)** GSEA analysis showing age-pos genes significantly enriched in severe COVID-19 patients (left), and age-neg genes were upregulated in healthy controls (right). Differentially expressed analysis was performed based on 7 severe patients during progression stage and 8 age-matched healthy controls. NES, normalized enrichment score. **(e)** Comparison of adjusted ssGSEA score based on age-pos genes (left) or age-neg genes (right) among healthy controls (n = 8), and age-matched mild-moderate (n = 10) and severe (n = 7) patients during progression stages. Statistical analysis was performed using the two-sided Kruskal-Wallis test (**b, c**) and the two-tailed Student’s t test (**b, c, e**), and adjusted using the BH correction method (**b-d**). Data are from GSE158055^1^.


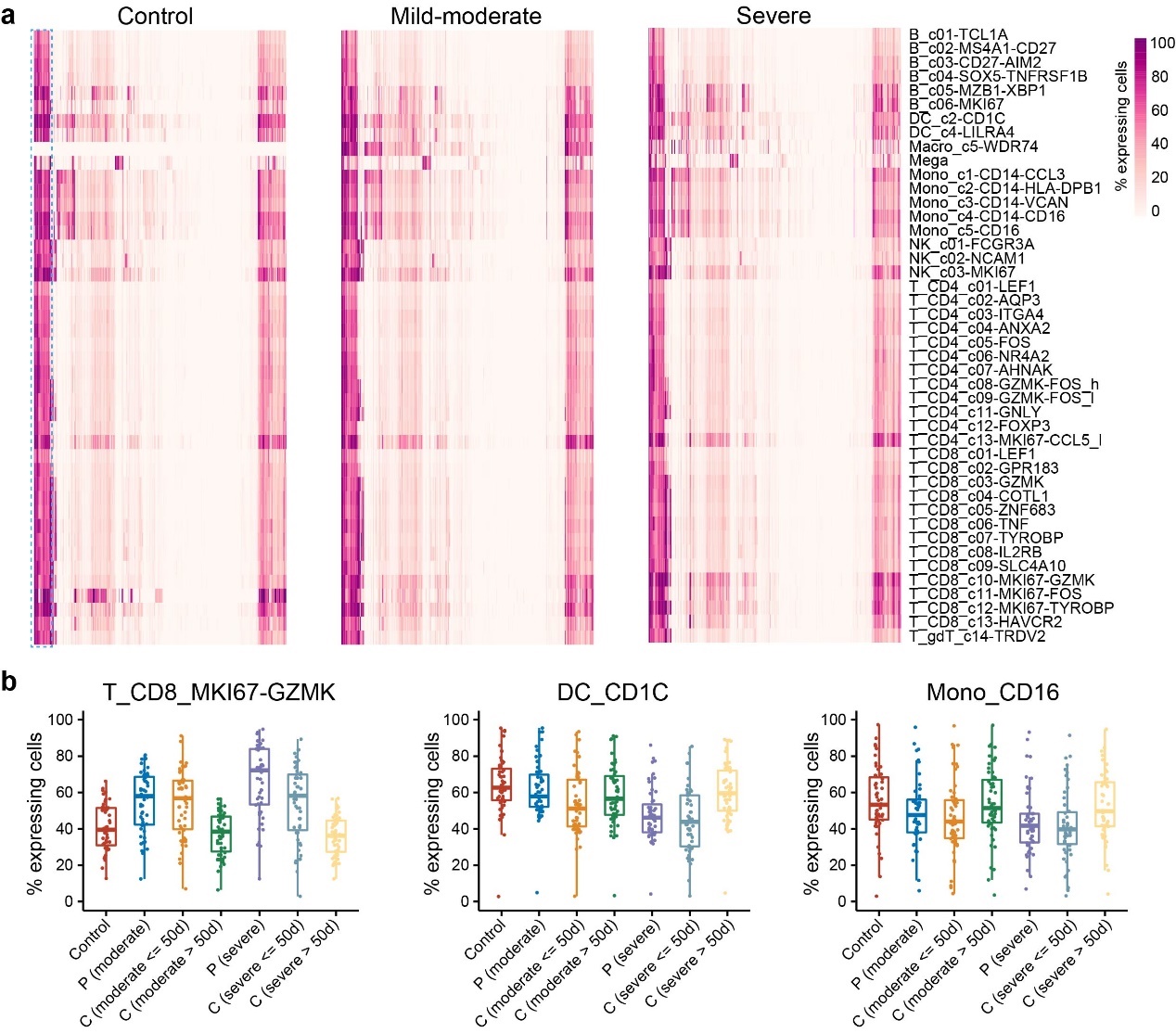


**Supplementary Figure 5. Comparison of single-cell type expression profiles of age-pos genes among patients with different severity.** **(a)** Heatmap showing the percentage of cells expressing each of the age-pos genes in healthy controls (left, n = 15), and mild-moderate (middle, n = 15) and severe patients (right, n = 12) during progression stages. Only fresh samples were used in this chapter. Dotted box showing the 51 highly enriched genes in almost all cell-types. Data are from GSE158055^1^. **(b)** Comparison of percentages of cells expressing 51 genes in three cell subsets among patient groups. According to the time of sample collection, patients at the convalescence stage were further divided into early (<= 50 days) and late (> 50 days) convalescence. P, progression; C, convalescence.


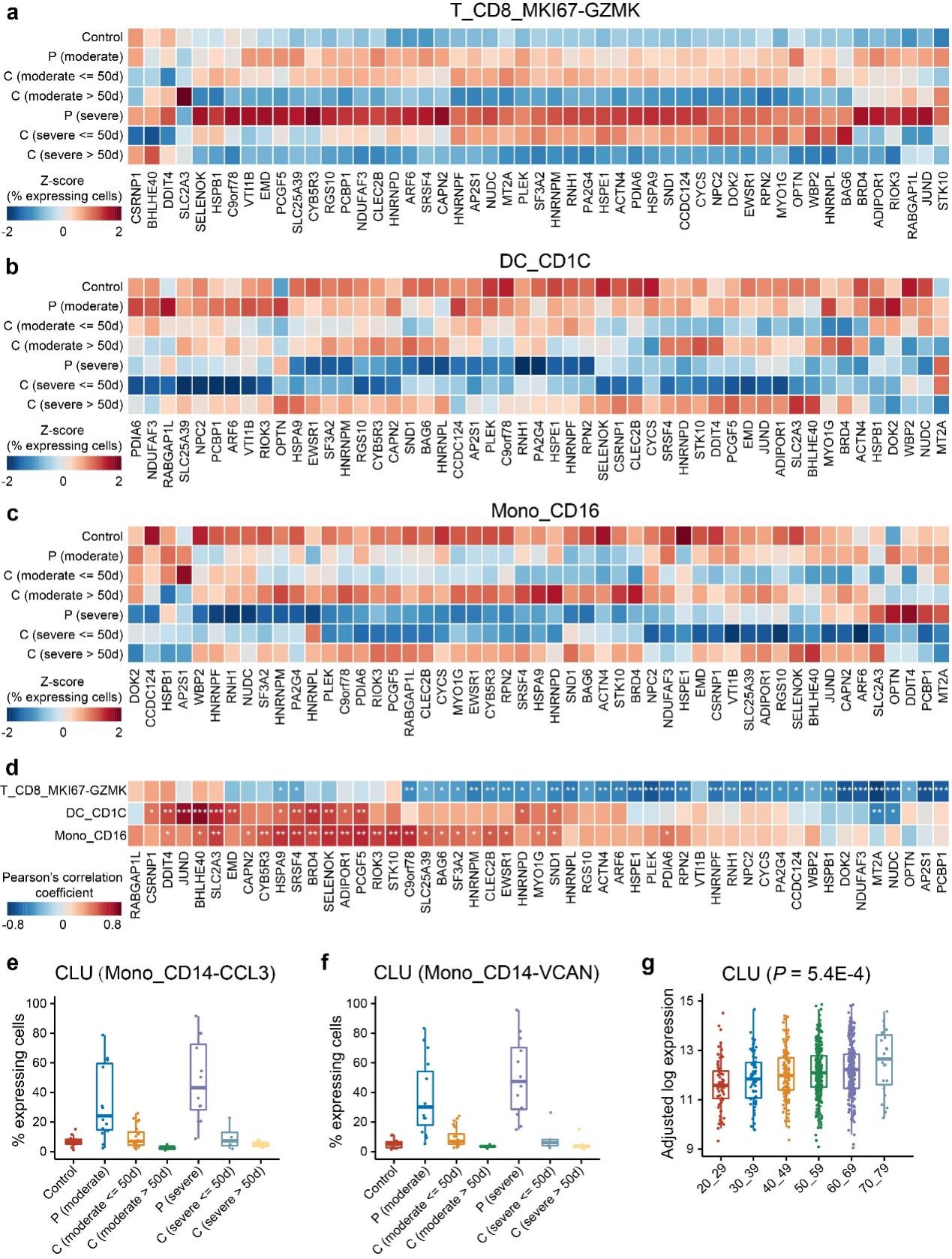


**Supplementary Figure 6. Single-cell type expression changes of some age-pos genes with patient severity and stages. (a-c)** Heatmap showing comparison of percentage of cells within T_CD8_c10-MKI67-GZMK (a), DC_c2-CD1C (b) or Mono_c5-CD16 (c) expressing each of 51 genes among patients with different disease severity and stages, scaled by gene across the different groups. Such 51 genes were from Supplementary Figure 5. Patients were grouped with the same as Supplementary Figure 5b. **(d)** The correlation between percentage of cells within each cell-type expressing each gene and sample time during the disease convalescence stage. Correlation coefficients and *P* values were calculated by the Pearson correlation method. **(e, f)** Comparison of percentage of cells within Mono_c1-CD14-CCL3 (e) or Mono_c3-CD14-VCAN (f) expressing *CLU* among different patient groups. **(g)** Comparison of *CLU* expression among different age groups. Expression were estimated by controlling sex and hardy scale. Statistical analysis was performed using the two-sided Kruskal-Wallis test **(g)** and adjusted using the BH correction **(d)**. **P*.adjust < 0.05, ***P*.adjust < 0.01, ****P*.adjust < 0.001. Data in **(a-f)** are from GSE158055^1^.


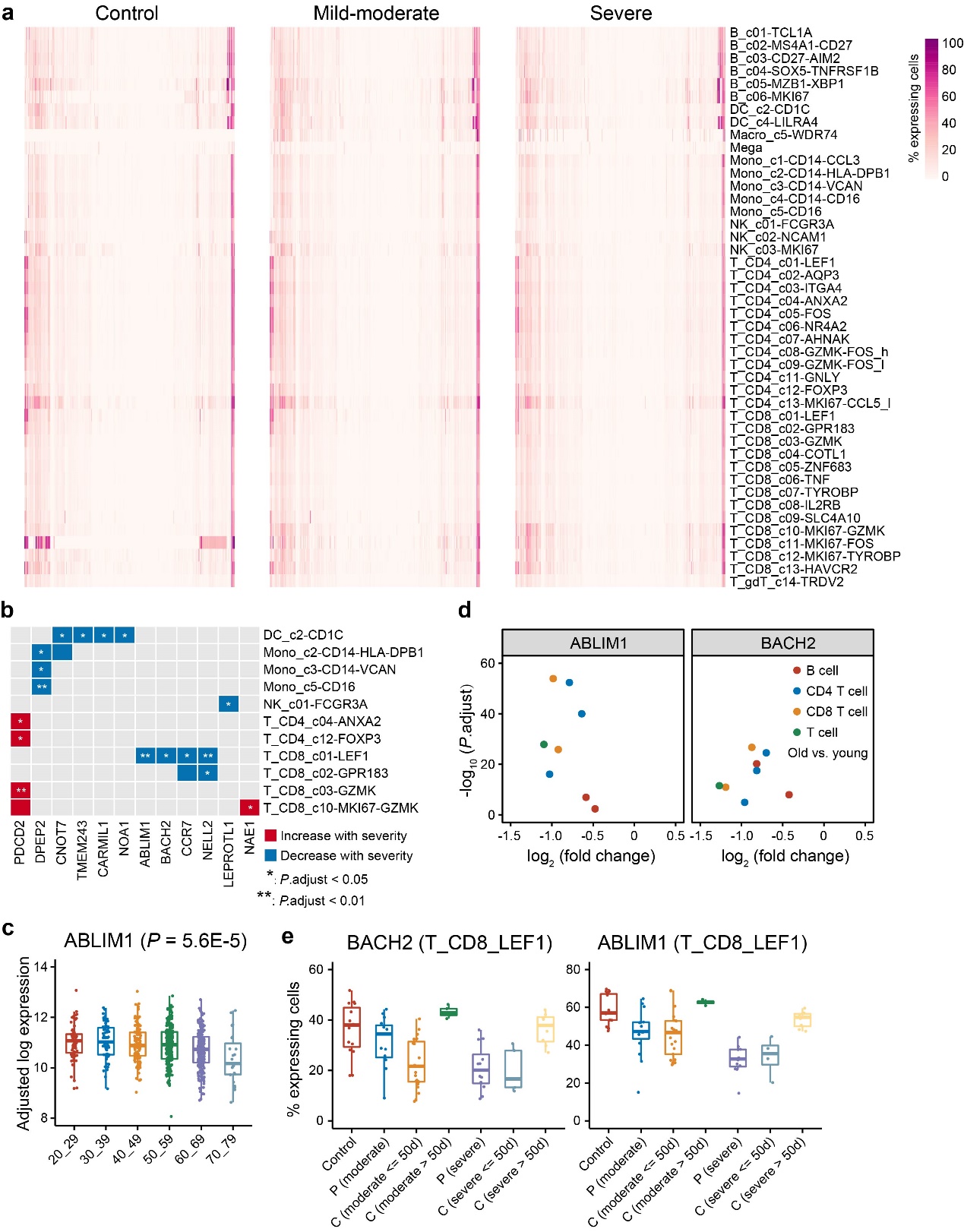


**Supplementary Figure 7. Comparison of single-cell type expression profiles of age-neg genes among patient with different severity. (a)** Heatmap showing the percentage of cells expressing each of the age-neg genes in healthy controls (left, n = 15), and mild-moderate (middle, n = 15) and severe patients (right, n = 12) during progression stages. **(b)** Heatmap showing the cell-types with significant difference in proportion of cells expressing some age-neg genes among healthy controls (n = 15), and mild-moderate (n = 15) and severe patients (n = 12) during progression stages. The three cut-off criteria were used to identify differential age-neg genes: (1) gene expression in > 30% cells within one cell-type from healthy controls or severe patients; (2) fold change >1.5 or < 0.67 after add 10% to percentages of cells expressing one gene between healthy controls and severe patients; (3) BH adjusted *P* < 0.05. The color indicates the trend, red represents the percentage of cells increased with severity, and blue denotes decreased with severity. **(c)** Comparison of *ABLIM1* expression among different age groups. Expression were estimated by controlling sex and hardy scale. **(d)** Comparison of *ABLIM1* and *BACH2* expression of main cell-types from human PBMCs between young healthy adults and aged healthy adults. **(e)** Comparison of percentage of cells within T_CD8_LEF1 expressing *BACH2* (left) and *ABLIM1* (right) among different patient groups. Statistical analysis was performed using the two-sided Kruskal-Wallis test **(b, c)** and adjusted using the BH correction (**b**). **P*.adjust < 0.05, ***P*.adjust < 0.01, ****P*.adjust < 0.001. Data in **(a, b, e)** are from GSE158055^1^. Data in **(d)** are from an Aging Atlas Consortium (https://ngdc.cncb.ac.cn/aging/index).


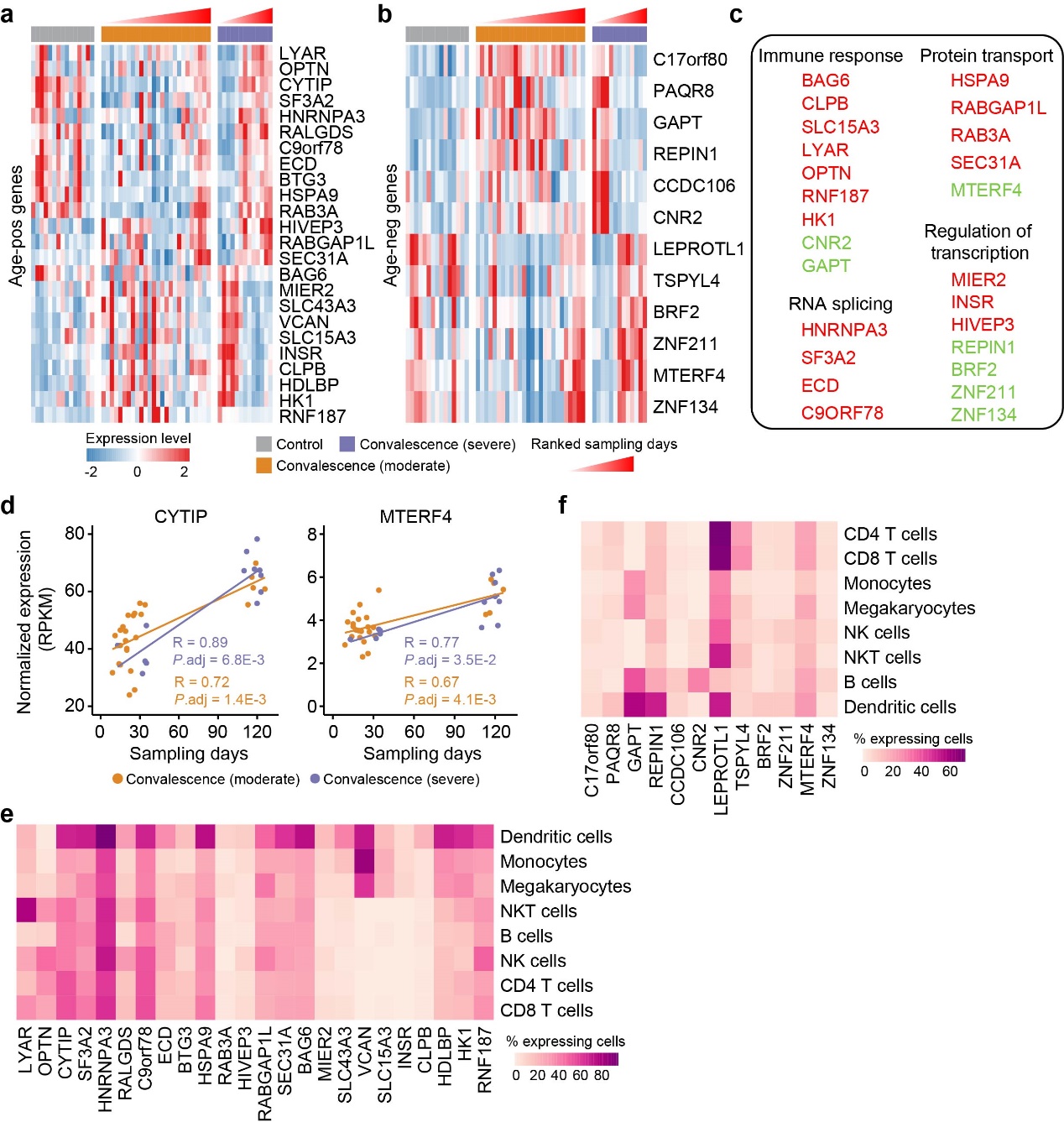


**Supplementary Figure 8. Persistent alteration of age-related gene expression at convalescent stage. (a, b)** Normalized expression profiles of selected age-pos genes (a) or age-neg genes (b) in healthy controls (n = 15), and mild-moderate (n = 26) and severe patients (n = 13) during convalescent stage. These genes expression was significantly correlated with sampling days (days after symptom onset) of severe patients in convalescent stage (BH adjusted *P* < 0.05). Triangle denotes sampling days were ranked from low to high in mild-moderate and severe groups. **(c)** Enriched pathways of age-related genes described in (a) and (b). Red and green represent age-pos genes and age-neg genes, respectively. **(d)** Pearson’s correlation of normalized expression of two age-related genes with sampling days (days after symptom onset) in mild-moderate (yellow) and severe patients (blue) during convalescent stage. **(e, f)** The percentage of cells expressing each of selected age-pos genes (e) and age-neg genes (f). Data in **(a, b, d)** are from GSE158055^1^. Data in **(e, f)** are from scRNA-seq profiles of healthy 5' PBMCs^2^.


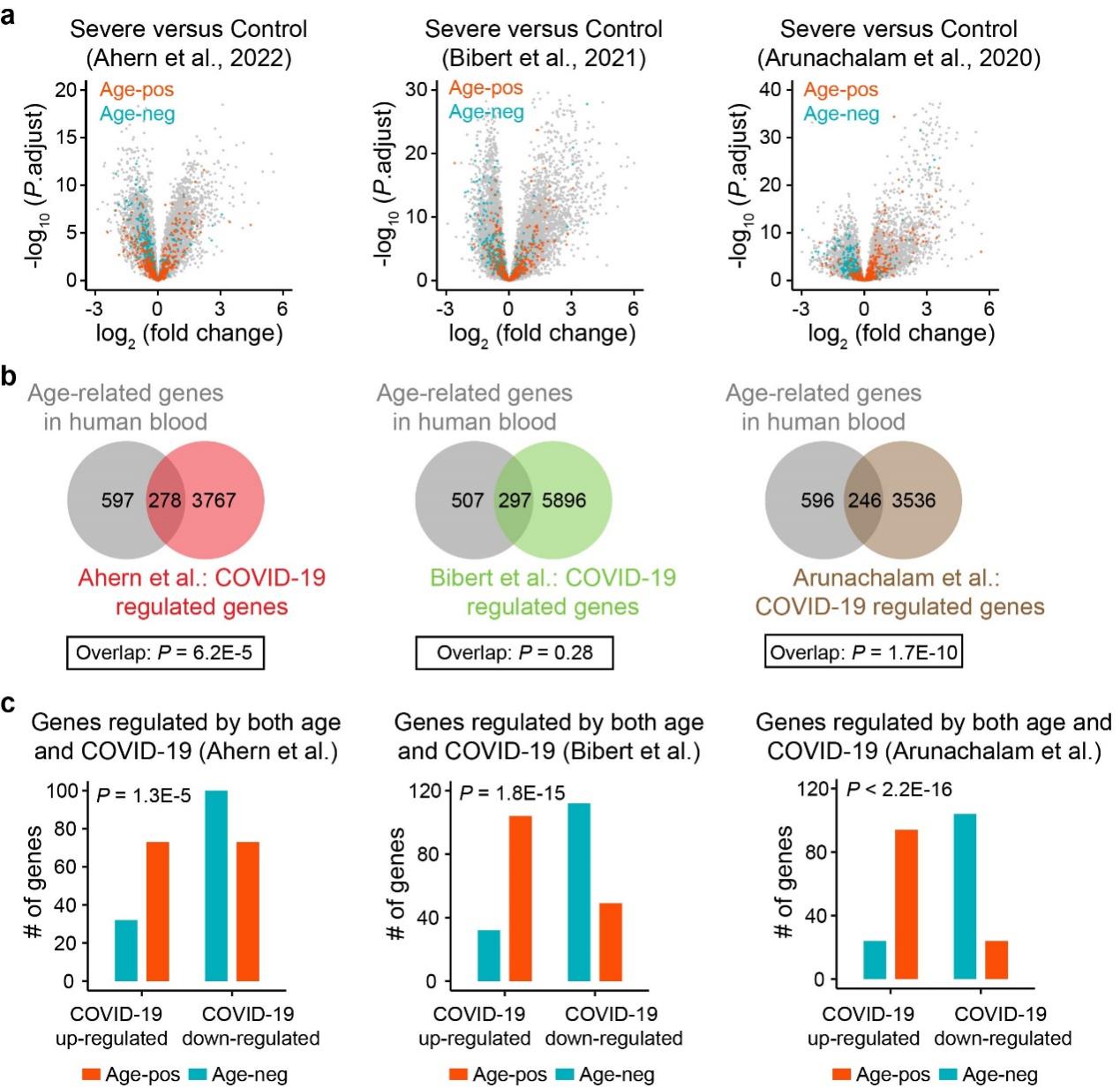


**Supplementary Figure 9. Impact of SARS-CoV-2 infection on age-related gene expressions. (a)** Volcano plots of differentially expressed genes between severe patients and healthy controls. Data were collected from three publicly independent studies^3-5^. Orange and blue represent age-pos genes and age-neg genes, respectively. **(b)** Venn diagrams showing the intersections between blood age-related genes and COVID-19 regulated genes from three independent studies^3-5^. **(c)** The intersections between the directionality of change in age-related gene expression with age and the directionality of change in COVID-19 regulated genes expression caused by SARS-CoV-2 infection across three datasets in (a). Statistical analysis was performed using the hypergeometric test **(b)** or two-sided Fischer’s exact test **(c)**.


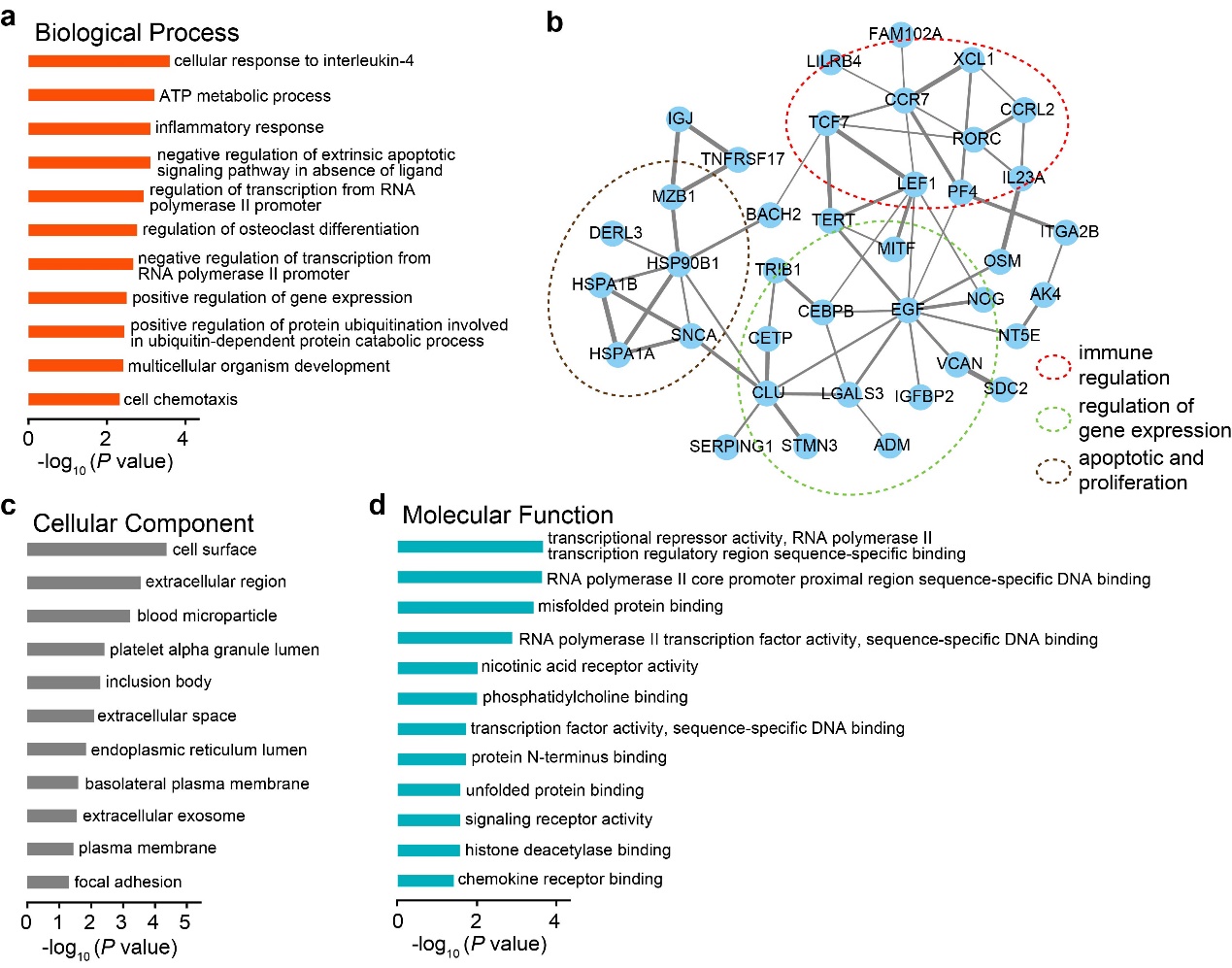


**Supplementary Figure 10. Functional enrichment analysis.** **(a)** Biological processes (BP) enrichment analysis of the 99 overlapping age-related genes that were regulated by SARS-CoV-2 infection in Fig. 1e. **(b)** The 99 genes identified in Fig. 1e were subjected to network analysis using the STRING database. The interaction networks were then imported into Cytoscape for visualization. (**c, d**) Cellular Component (CC) (c) and Molecular Function (MF) (d) analysis of the 99 overlapping genes using DAVID database.


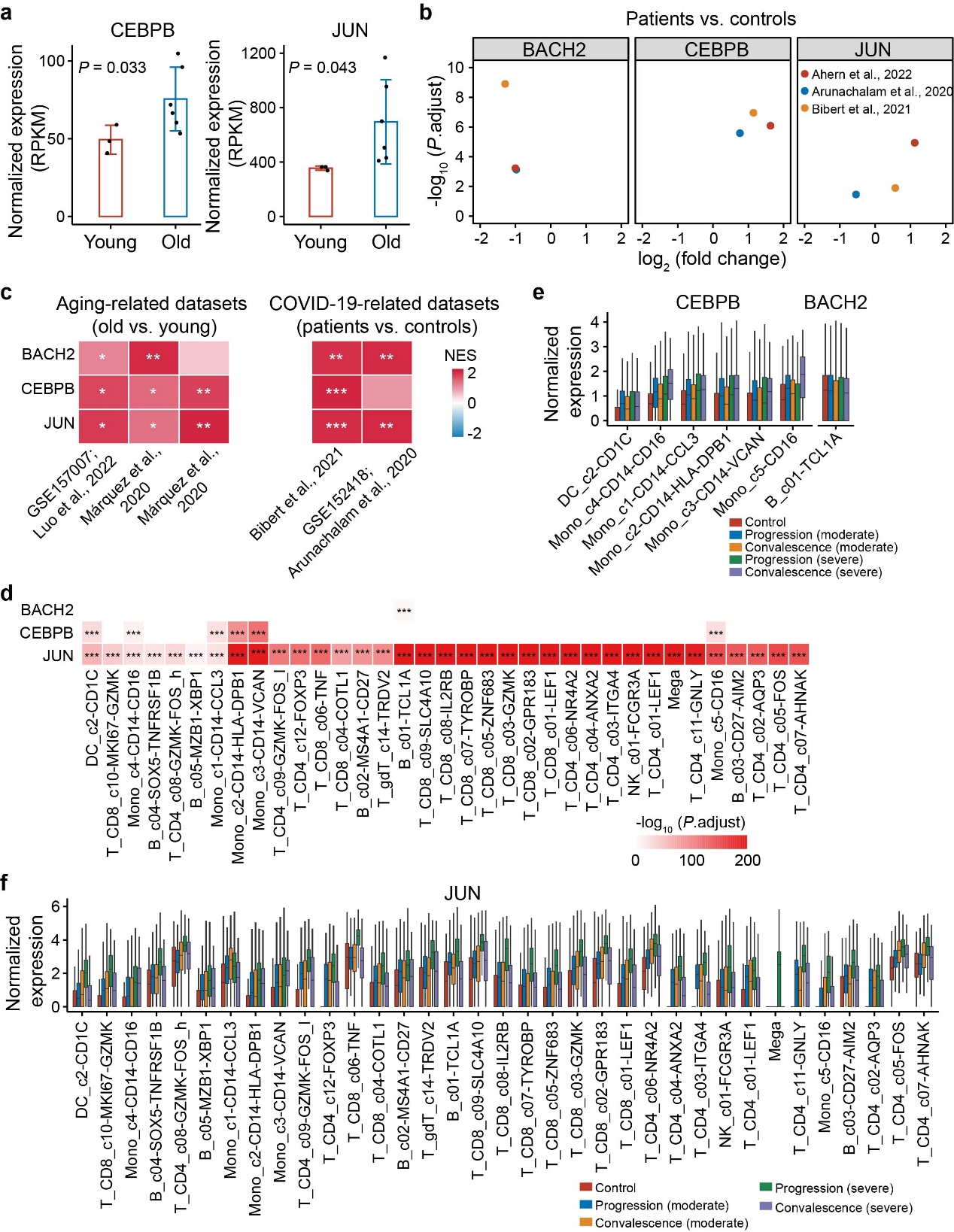


**Supplementary Figure 11. Comparative analysis of three aging-related transcription factors expression between age groups or patient groups. (a)** Validation of differential expression of *CEBPB* and *JUN* between old and young healthy adults in one independent study. Sample sizes of n = 3 and 6 for young and old groups, respectively. Data were presented as the mean ± s.d. Data are from GSE157007^7^. **(b)** Comparison of *BACH2*, *CEBPB* and *JUN* expression between COVID-19 patients and healthy controls in three studies^3-5^. (**c**) GSEA results based on top 100 putative target genes of each transcription factor (*BACH2*, *CEBPB* and *JUN*). Differential expression analysis was performed between old and young healthy adults (left), or COVID-19 patients and healthy controls (right). Aging-related datasets and COVID-19-related datasets were collected from independent studies, respectively^3,4,7,9^. Top 100 putative target genes were collected from the Cistrome platform. NES, normalized enrichment score. **(d)** Comparison of three transcription factors expression in each cell-type among healthy controls (n = 8), and age-matched mild-moderate (n = 10) and severe patients (n = 7) during progression stages. Data are from GSE158055^1^. **(e, f)** Comparison of normalized expression of *CEBPB* (e, left), *BACH2* (e, right) and *JUN* (f) in some cell-types detected in (d) among healthy controls (n = 8), progression (moderate, n = 10), convalescence (moderate, n = 24), progression (severe, n = 7) and convalescence (severe n = 10) patients. Data are from GSE158055^1^. Statistical analysis was performed using the two-tailed Student’s *t* test (**a**) or two-sided Kruskal-Wallis test **(d),** and adjusted using the BH correction **(c, d)**. **P*.adjust < 0.05, ***P*.adjust < 0.01, ****P*.adjust < 0.001.

**
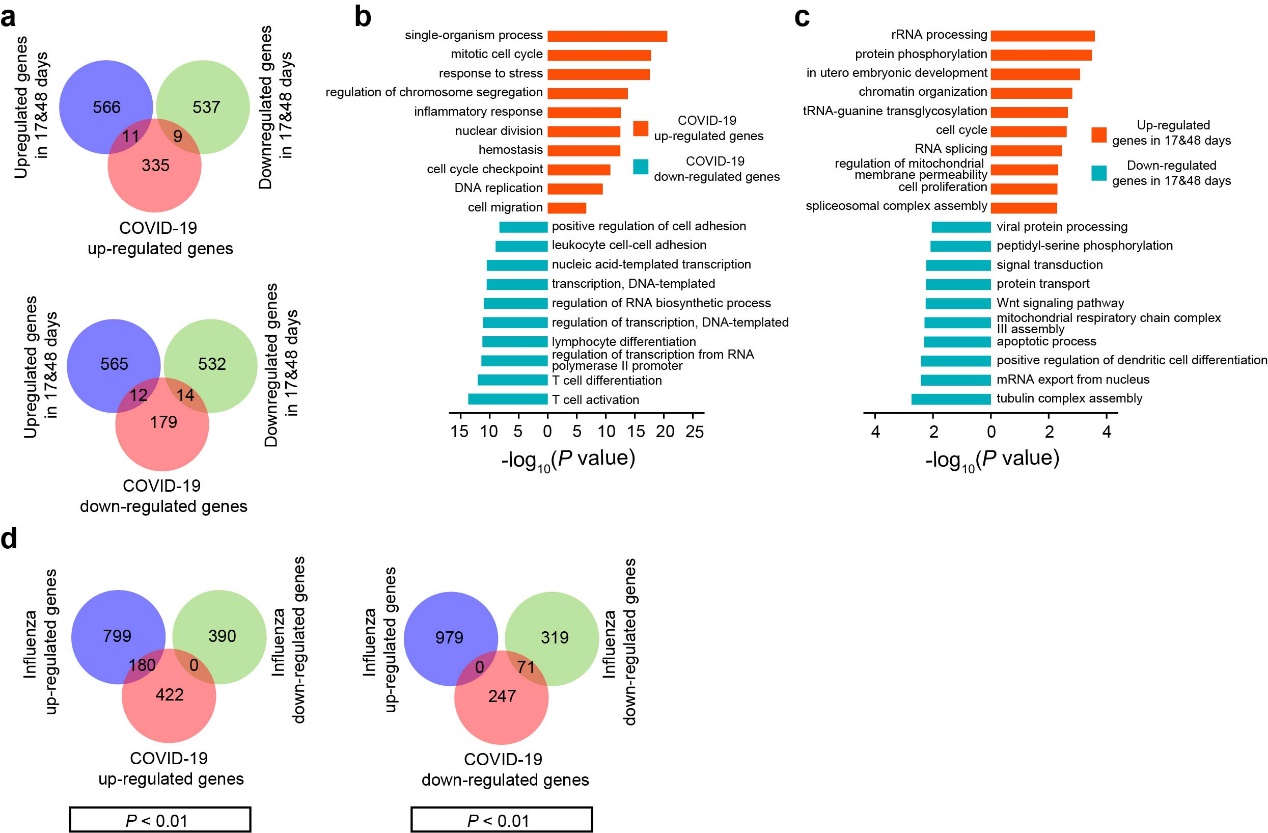
**

**Supplementary Figure 12. Comparison of transcriptional changes caused of SARS-CoV-2 infection with other viruses.** **(a)** Venn diagram showing the intersections of COVID-19 up-regulated genes and Zika regulated genes. Data are from GSE123541^29^. **(b)** DAVID gene ontology (GO) analyses of COVID-19 up-regulated genes (orange) and COVID-19 down-regulated genes (blue). **(c)** DAVID gene ontology analyses of up-regulated genes in 17&48 days (orange) and down-regulated genes in 17&48 days after acute Zika virus infection (blue). **(d)** Venn diagram showing the intersections of COVID-19 regulated genes and Influenza regulated genes (hypergeometric test).

**
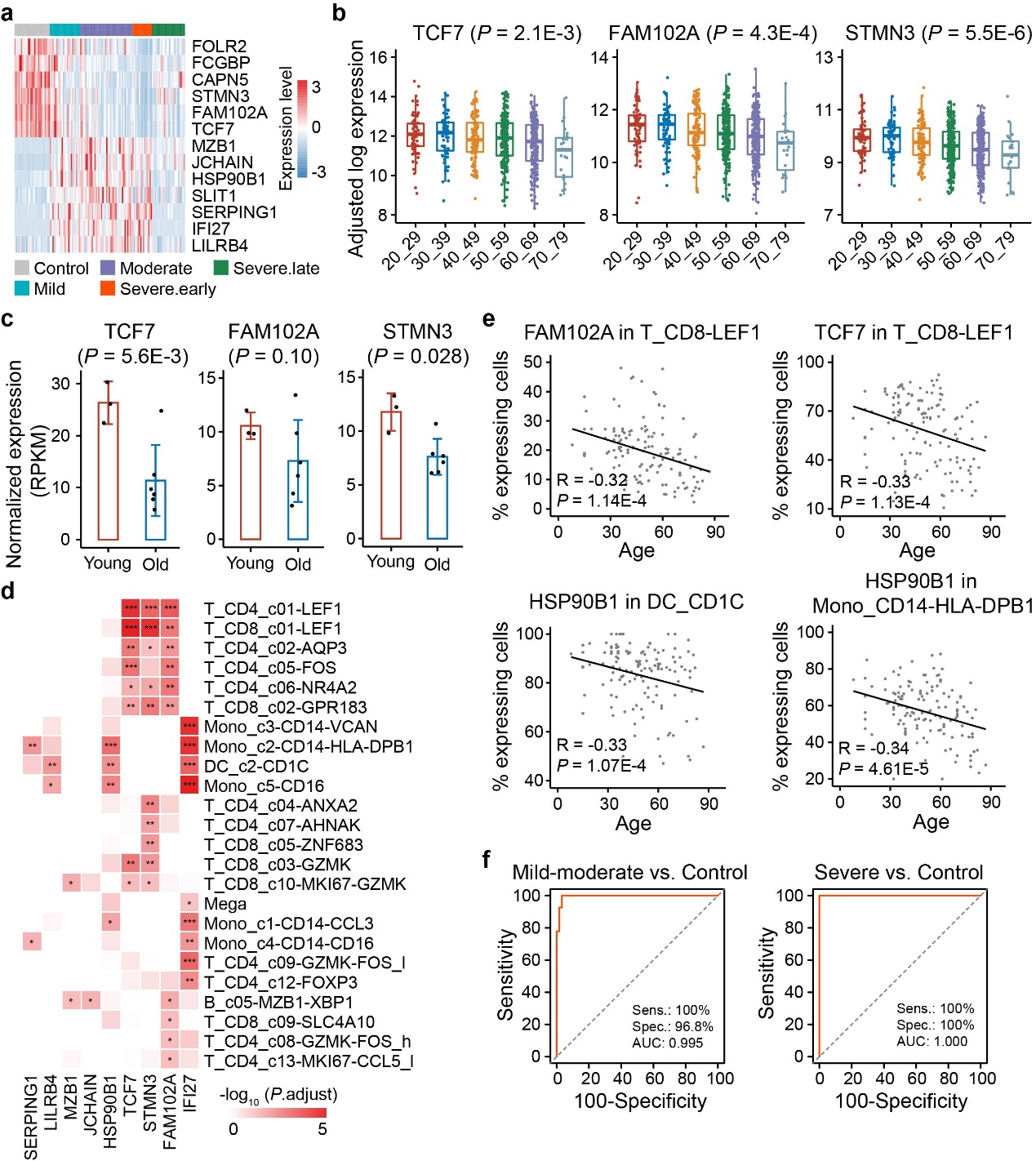
**

**Supplementary Figure 13. The 13 age-related genes that were regulated by SARS-CoV-2 infection for a severity scoring model. (a)** Comparison of normalized expression profiles of the 13 genes among healthy controls (n = 27), and mild (n = 23), moderate (n = 40), severe (early, n = 15) and severe (late, n = 25) patients. Early severe patients were sampled within the first 7 days in hospital (“Severe.early”), while late severe patients (“Severe.late”) were sampled later. **(b)** Comparison of *TCF7*, *FAM102A* and *STMN3* expression among different age groups. Expression was estimated by controlling sex and Hardy scale. **(c)** Validation of differential expression of *TCF7*, *FAM102A* and *STMN3* between old and young healthy adults in one independent study. Sample sizes of n = 3 and 6 for young and old groups, respectively. Data were presented as the mean ± s.d.. **(d)** Heatmap showing the cell-types with significant difference in proportion of cells expressing 13 genes among healthy controls (n = 20), and mild-moderate (n = 18) and severe patients (n = 38) during progression stages. **(e)** Association between age and the percentage of cells expressing *FAM102A* or *TCF7* in T_CD8-LEF1, and HSP90B1 in DC_ CD1C or Mono_CD14-HLA-DPB1 (Pearson’s correlation coefficients). **(f)** ROC curves of the severity score in distinguishing mild-moderate (left, n = 63) or severe patients (right, n = 40) from healthy controls (n = 27). Statistical analysis was performed using the two-sided Kruskal-Wallis test (**b, d**) or two-tailed Student’s t test (**c**), and adjusted using the BH correction (**d**). **P.*adjust < 0.05, ***P.*adjust < 0.01, ****P.*adjust < 0.001. Data in **(a, f)** are from Bibert et al.^3^. Data in **(c)** are from GSE157007^7^. Data in **(d, e)** are from GSE158055^1^.

**Data S1. (separate file)**

List of age-related genes and cell-type characteristics

**Data S2. (separate file)**

Unadjusted and adjusted ssGSEA score

**Data S3. (separate file)**

Cell-type expression of age-related genes in different patient groups

**Data S4. (separate file)**

Differential expression analysis between healthy controls and COVID-19 patients, list of age-related genes that were perturbed by SARS-CoV-2 infection, and top 100 putative target genes for transcription factor
